# Supplementary material for: Content-rich biological network constructed by mining PubMed abstracts
Source: BMC Bioinformatics. 2004 Oct 8;5:147. doi: 10.1186/1471-2105-5-147 (PMC528731; doi:10.1186/1471-2105-5-147)
Supplement: Additional File 2 — The original results of the above study (non-essential files are deleted to keep the file size under the limit set by BMC bioinformatics). [file 1471-2105-5-147-S2.bz2 › chilibotAdditionalFile2/dip05/732ID11983079E18090/html/HSCA_HSCB.html]

 


 **HSCA** and **HSCB** 
  
Found 16 abstracts in PubMed, retrieved 05.  
 

 What does Google say? 
 PDF only 
| .edu only 

---

**Interactive relationship** (e.g. stimulation, inhibition, etc)

**Neutral relationship**- Hsc66  [ **HSCA** ]   **HscA**  and Hsc20  [ **HSCB** ]   **HscB**  from Escherchia coli comprise a specialized chaperone system that selectively binds the iron sulfur cluster template protein IscU.  Ref: 12871959 J Biol ChemJ Biol Chem,
- Contributions of the LPPVK motif of the iron sulfur template protein IscU to interactions with the Hsc66  [ **HSCA** ]  Hsc20  [ **HSCB** ]  chaperone system.  Ref: 12871959 J Biol ChemJ Biol Chem,
- These results indicate that residues in the LPPVK motif are important for IscU interactions with Hsc66  [ **HSCA** ]  but not for the ability of Hsc20  [ **HSCB** ]  to target IscU to Hsc66  [ **HSCA** ] .  Ref: 12871959 J Biol ChemJ Biol Chem,
- In accord with the previous in vitro binding and kinetic experiments, interactions were observed for the combinations of IscS and IscU, IscU and  **HscB** , IscU and  **HscA** , and  **HscB**  and  **HscA** .  Ref: 11983079 J Biochem Tokyo , 2002
- A charge mutant containing the motif sequence of NifU, IscU V102E, did not bind the ATP or ADP states of Hsc66  [ **HSCA** ]  but did bind Hsc20  [ **HSCB** ]  and weakly stimulated Hsc66  [ **HSCA** ]  ATPase in the presence of the co chaperone.  Ref: 12871959 J Biol ChemJ Biol Chem, 2002

**Non-interactive relationship** (e.g. studied together, co-existance, homology, etc.)

- When ATPase stimulatory activity was assayed in the presence of the co chaperone Hsc20  [ **HSCB** ] , each of the mutants displayed enhanced binding affinity, but the P101A and V102A mutants exhibited decreased ability to maximally simulate Hsc66  [ **HSCA** ]  ATPase.  Ref: 12871959 J Biol ChemJ Biol Chem, 2002
- In 56 available genomes we identified two genes with identical phylogenetic distributions to the frataxin cyaY gene  **hscA**  and  **hscB**  JAC1.  Ref: 11689493 Hum Mol Genet, 2001
- Our results indicate that Hsc66  [ **HSCA** ]  and Hsc20  [ **HSCB** ]  recognize distinct regions of IscU and further suggest that Hsc66  [ **HSCA** ]  will not bind LPPVK motifs with high affinity in vivo unless they are in the context of native IscU and can be directed to Hsc66  [ **HSCA** ]  by Hsc20  [ **HSCB** ] .  Ref: 11994302 J Biol Chem, 2002
- Thus, the sequential association and dissociation among the IscS, IscU, IscA,  **HscB** ,  **HscA** , Fdx, and ORF3 proteins may be a critical process in the assembly of Fe S clusters.  Ref: 11983079 J Biochem Tokyo , 2002
- Only a single substrate, the Fe S template protein IscU, has been identified for the Hsc66  [ **HSCA** ]  Hsc20  [ **HSCB** ]  system, but the mechanism by which Hsc66  [ **HSCA** ]  selectively binds IscU is unknown.  Ref: 11994302 J Biol Chem, 2002
- Hsc66  [ **HSCA** ]  and Hsc20  [ **HSCB** ]  comprise a specialized chaperone system important for the assembly of iron sulfur clusters in Escherchia coli.  Ref: 11994302 J Biol Chem, 2002
- The proteins encoded by  **hscA**  and  **hscB** , the chaperone HSP66 and the co chaperone HSP20, have been shown to be required for the synthesis of 2Fe 2S clusters on ferredoxin in proteobacteria.  Ref: 11689493 Hum Mol Genet, 2001
- JAC1, an ortholog of  **hscB** , and SSQ1, a paralog of  **hscA** , have been shown to be required for iron sulfur cluster assembly in mitochondria of Saccharomyces cerevisiae.  Ref: 11689493 Hum Mol Genet, 2001
- These findings are consistent with the role in Fe S biogenesis previously proposed for the bacterial Hsp70 Hsc66  [ **HSCA** ]  and J protein Hsc20  [ **HSCB** ]  that interact with the bacterial Isu homologue IscU.  Ref: 12756240 J Biol Chem< MedlineTA>J Biol Chem, 2001
- Unlike native IscU the synthetic peptide is not bound by Hsc20  [ **HSCB** ]  and does not synergistically stimulate Hsc66  [ **HSCA** ]  ATPase activity with Hsc20  [ **HSCB** ] .  Ref: 11994302 J Biol Chem, 2002
